# The pregnancy outcomes in patients with stage 3–4 chronic kidney disease and the effects of pregnancy in the long-term kidney function

**DOI:** 10.1007/s40620-018-0509-z

**Published:** 2018-07-19

**Authors:** Yingdong He, Jing Liu, Qingqing Cai, Jicheng Lv, Feng Yu, Qian Chen, Minghui Zhao

**Affiliations:** 1 Department of Obstetrics and Gynecology, Peking University, First Hospital, Beijing, 100034 People’s Republic of China; 2 Renal Division, Department of Medicine, Peking University, First Hospital, Peking University Institute of Nephrology, Beijing, People’s Republic of China

**Keywords:** Chronic kidney disease, Pregnancy, Renal disease progression

## Abstract

**Objective:**

To investigate the pregnancy outcomes for patients with stage 3–4 chronic kidney disease (CKD) and the effects of pregnancy on kidney function.

**Methods:**

Clinical data of pregnant women with CKD in the Peking University First Hospital between January 1st 2005 and October 1st 2016 were retrospectively analysed. The pregnancy outcomes of patients with different stages of CKD were compared. Patients with stage 3–4 CKD were followed up by telephone interview, and non-pregnant patients with stage 3–4 CKD were selected using the propensity score method to analyse the effects of pregnancy on kidney function.

**Results:**

A total of 293 women with 300 pregnancies met the study criteria. There were 30 cases of stage 3–4 CKD. The incidence of adverse pregnancy outcomes of patients with stage 3–4 CKD was significantly higher than that with stage 1 CKD. The mean postpartum follow-up time of pregnant patients with CKD was 49.0 ± 33.1 months. A total of 26 cases of stage 3–4 CKD were followed up. During the follow-up period, 8 patients progressed to ESRD. A total of 28 non-pregnant patients with stage 3–4 CKD were selected as the control group. The results of multivariate analysis revealed that pregnancy did not increase the risk of deterioration of kidney function.

**Conclusion:**

Patients with stage 3–4 CKD in early pregnancy had a significantly increased risk of adverse pregnancy outcomes. Pregnancy itself did not seem to accelerate kidney disease progression in patients with stage 3–4 CKD.

## Introduction

Chronic kidney disease (CKD) is a global health issue. The Kidney Disease Outcomes Quality Initiative (KDOQI) workshop of the National Kidney Foundation of the USA released guidelines in 2002 to update the definition of CKD [1]. The Kidney Disease: Improving Global Outcomes (KDIGO) organization further improved and expanded the 2002 guidelines [2]. With the implementation of these guidelines, the incidence of CKD has increased annually, and approximately 3% women of childbearing age have CKD [3, 4].

Many studies have evaluated the pregnancy outcomes of women with CKD and the effects of pregnancy on the progression of kidney diseases. They show that although the incidence of adverse pregnancy outcomes is significantly increased with respect to healthy women, patients with essentially normal renal function usually have acceptable pregnancy outcomes, and pregnancy does not have an obvious effect on kidney disease progression, although proteinuria may be a marker of renal function worsening [5–10]. However, patients with moderate to severe renal impairment at early pregnancy have a significantly increased risk of adverse pregnancy outcomes, and pregnancy may accelerate kidney disease progression in these patients. Besides renal function, many other factors determine pregnancy and long-term renal outcomes of patients with stage 3–4 CKD, including baseline blood pressure, proteinuria and treatment. As in some patients CKD is first diagnosed during pregnancy, if we can predict pregnancy outcomes and the effects of pregnancy on renal function using clinical indicators in early pregnancy, we can provide better advice to these patients, especially for patients with stage 3–4 CKD. At present, whether the prognosis of stage 3–4 CKD is influenced by pregnancy remains controversial. Thus, in this study, we aimed to evaluate the effect of pregnancy on kidney disease progression in stage 3–4 CKD and explore clinically feasible risk factors for adverse pregnancy outcomes. We investigated the following issues: (1) the pregnancy outcomes of patients with stage 3–4 CKD; (2) risk factors for adverse pregnancy outcomes in patients with CKD and (3) the effects of pregnancy on the deterioration of renal function in stage 3–4 CKD patients.

## Materials and methods

### Study subjects

Of 362 pregnant patients with CKD who received prenatal care in the Department of Obstetrics and Gynecology of Peking University First Hospital, Peking, China between January 1 2005 and October 1 2016, 57 patients were transferred to our hospital in the 2nd or 3rd trimester and we did not have their information in early pregnancy while 12 patients delivered in other hospitals. A total of 293 pregnant women with CKD and complete pregnancy and childbirth data were eligible for the study. If a patient had multiple occurrences of pregnancy and childbirth, each pregnancy was regarded as a separate case. The study was performed in compliance with the Declaration of Helsinki and approved by the local ethical committee of Peking University First Hospital. All participants provided written informed consent.

### Study methods

Medical data for patients during pregnancy were retrieved, and clinical data for study subjects were retrospectively analyzed.

1. *Collection of clinical data* The following clinical data were collected: age, kidney disease history, therapy in early pregnancy (including antihypertensive drugs and immunosuppressive agents), pathological results of renal biopsy, baseline blood pressure, body weight, body height, serum creatinine (Scr), and 24-h proteinuria. Mean arterial pressure (MAP) was calculated as (systolic pressure + 2 × diastolic pressure)/3. Body mass index (BMI) was calculated based on height and body weight. According to age and Scr level, the estimated glomerular filtration rate (eGFR) was calculated using the modification of diet in renal disease (MDRD) equation [2]. Pregnancy outcomes included gestational age at birth, the development of hypertensive disorders during pregnancy, delivery method, neonatal birth weight, and maternal and neonatal death.
2. *CKD diagnosis and staging* The diagnosis of CKD was based on a history of CKD, clinical examination, renal function tests, 24-h urinary protein excretion, renal ultrasound and renal biopsy. Renal disease had to be evident before 20 weeks gestation for patients to be eligible for this study. For patients who underwent renal biopsy, the type of CKD was determined based on biopsy results; for the other patients, CKD type was determined based on clinical diagnosis. The staging of kidney diseases was based on the eGFR level [2].
3. *Evaluation of pregnancy outcomes* Adverse pregnancy outcomes included maternal death, severe preeclampsia, renal failure, early preterm birth, very-low birth-weight infants, fetal loss after 20 weeks of pregnancy, and neonatal death. The diagnostic criteria for severe preeclampsia in patients with normal blood pressure and no proteinuria were based on the 2013 Hypertension in Pregnancy Guidelines of the American College of Obstetricians and Gynecologists (ACOG) [11]. In women who had proteinuria but no hypertension in early pregnancy, the diagnosis of preeclampsia required the presence of thrombocytopenia, a sudden increase in proteinuria (either 5 times the baseline value or twice the baseline value if the baseline exceeded 2 g/24 h), or hypertension accompanied by severe headaches, epigastric pain, or a serum aspartate aminotransferase concentration > 70 U/l. In women who had both hypertension and proteinuria in early pregnancy, the diagnosis of preeclampsia required any one of the following criteria: elevated concentration of serum aspartate aminotransferase (> 70 U/l), thrombocytopenia, or worsening hypertension (systolic blood pressure ≥ 140 mmHg with a rise of at least 30 mmHg or diastolic blood pressure ≥ 90 mmHg with a rise of at least 15 mmHg) accompanied by severe headaches or epigastric pain [12, 13]. The diagnostic criterion for early preterm birth was gestational age at birth of less than 34 weeks [6–9]. The diagnostic criterion for very-low-birth-weight infants was neonatal birth weight < 1500 g [6–9]. End-stage renal disease (ESRD) was defined as eGFR < 15 ml/min/1.73 m^2^ or initiation of renal replacement therapy [2].
4. *Renal function follow-up* All patients were followed up via telephone interview between October 2015 and October 2016. Clinical endpoint events, including progression to ESRD and the need for dialysis treatment, were recorded.
5. *Controls* To better evaluate the effects of pregnancy on renal function in patients with stage 3–4 CKD, non-pregnant patients with stage 3–4 CKD from the Chinese immunoglobulin (Ig)A nephropathy database (http://ckd.edc-china.com.cn/) were selected as the control group. The patients were matched by age, gender, and baseline values of Scr, 24-h proteinuria, and MAP. The occurrence of renal failure was compared between pregnant and non-pregnant patients with stage 3–4 CKD.

### Statistical analyses

Depending on the distribution, baseline and follow-up data were compared using nonparametric tests or t tests. Continuous variables are presented as mean ± standard deviation (SD) or median and range. The relationships between patient variables and adverse pregnancy outcomes were assessed using logistic regression analysis. Relevant variables significantly associated with adverse pregnancy outcomes and composite kidney failure events in the univariate analysis were included in the multivariate models. Kaplan–Meier analysis was used to compare renal survival in the pregnant and non-pregnant groups. Time-dependent Cox regression was used to evaluate independent predictors of composite kidney failure events. The power was calculated using Power and Sample Size Calculations Software (version 3.0, http://biostat.mc.vanderbilt.edu/PowerSample Size). The Bonferroni correction for multiple comparisons was used. To better evaluate the effects of pregnancy on renal function in patients with stage 3–4 CKD, the propensity score method was used. Participants were matched using the greedy algorithm with 1:1 pairing. The calliper size was set at 0.025 × SD of the logit of the propensity scores. Balanced baseline covariates in the two groups were confirmed by paired comparison tests. A two-sided p value < 0.05 indicated statistical significance. SPSS 16 (IBM, Armonk, NY, USA) and STATA 11 (StataCorp, College Station, TX, USA) were used for statistical analyses.

## Results

### Comparison of baseline clinical data for pregnant patients with CKD

This study enrolled 293 patients with CKD diagnosed before or during pregnancy and included a total of 300 pregnancies. Thirty (10%) patients presented with stage 3–4 CKD. In early pregnancy, patients with more advanced CKD had a higher MAP and 24-h proteinuria. Regarding the diagnostic methods and types of kidney disease, 142 patients had undergone a renal biopsy, which revealed 113 cases of IgA nephropathy, 4 of membranous nephropathy, 11 of lupus nephritis, 9 of anaphylactoid purpura nephritis, 3 of diabetic nephropathy, and 2 of antineutrophil cytoplasmic antibody (ANCA)-associated systemic vasculitis. In the remaining 151/293 patients who did not undergo renal biopsy, CKD was diagnosed according to clinical presentation: 118 cases of chronic nephritis, 22 of nephrotic syndrome, 6 of diabetic nephropathy, and 5 of lupus nephritis. In the 30 patients with stage 3–4 CKD, 23 had received renal biopsy, which revealed 20 cases of IgA nephropathy, 1 case of membranous nephropathy, 1 case of anaphylactoid purpura nephritis, and 1 case of ANCA-associated systemic vasculitis. In the remaining 7/30 patients who did not undergo renal biopsy, there were 5 cases of chronic nephritis, and 2 of nephrotic syndrome. Baseline clinical data for CKD patients at different stages are shown in Table 1.

**Table 1:** Clinical characteristics of pregnant women with CKD

	Stage 1 (n = 197)	Stage 2 (n = 73)	Stages 3–4 (n = 30)	t/χ^2 α^	p^α^	t/χ^2β^	p^β^
Gestational time at baseline (weeks)	9.1 ± 2.9	8.7 ± 3.1	10.3 ± 4.9	0.82	0.37	1.36	0.18
Age (years)	30.7 ± 3.8	32.0 ± 3.5	31.4 ± 4.1	2.48	0.01	0.42	0.68
BMI (kg/m^2^)	23.5 ± 4.6	23.9 ± 3.9	23 ± 3.3	0.64	0.53	0.51	0.61
Serum creatinine (µmol/l)	51.9 ± 9.2	75.6 ± 8.4	157.1 ± 147.3	19.23	< 0.001	4.06	< 0.001
eGFR (ml/min/1.73 m^2^)	126.1 ± 31.4	78.2 ± 7.6	43.9 ± 13.2	19.83	< 0.001	25.00	< 0.001
MAP (mmHg)	83.6 ± 9.2	88.0 ± 12.1	91.5 ± 8.5	3.21	< 0.001	4.40	0.01
Proteinuria (g/day)	0.8 ± 1.3	0.9 ± 0.9	3.2 ± 2.8	0.35	0.73	4.58	< 0.001
≤ 1 [n (%)]^a^	143 (72.6)	50 (68.5)	9 (30)	0.44	0.51	21.35	< 0.001
1–2 [n (%)]^a^	30 (15.2)	14 (19.2)	1 (3.3)	0.61	0.42	2.20	0.14
> 2 [n (%)]^a^	24 (12.2)	9 (12.3)	20 (67.7)	0.001	0.97	49.50	< 0.001
HTN [n (%)]^a^	20 (10.2)	13 (17.8)	6 (20)	2.91	0.09	1.61	0.2
Therapy in early pregnancy [n, (%)]^a^	20 (10.2)	8 (11.0)	6 (20)	0.04	0.85	1.61	0.2

Statistical significance at p = 0.025 after Bonferroni correction for multiple comparisons

Values are expressed as mean ± standard deviation or number and percentage

*CKD* chronic kidney disease, *BMI* body mass index, *eGFR* estimated glomerular filtration rate, *MAP* mean arterial pressure, *HTN* hypertension

^a^Ratio of CKD patients at the corresponding stage

^α^CKD stage 2 compared to CKD stage 1

^β^CKD stage 3–4 compared to CKD stage 1

### Comparison of pregnancy outcomes of CKD patients at different stages

The pregnancy outcomes of patients with stage 2 or stage 1 CKD were not significantly different. However, the live birth rate, mean gestational age at birth, and neonatal birth weight were significantly lower in the group of pregnant patients with stage 3–4 CKD than in the group of pregnant patients with stage 1 CKD (p < 0.01). In addition, the incidence of preeclampsia, fetal and neonatal mortality, low-birth-weight infants and the preterm birth rate were significantly higher for pregnant patients with stage 3–4 CKD than for pregnant women with stage 1 CKD (p < 0.01). The pregnancy outcomes of patients with different stages of CKD are shown in Table 2.

**Table 2:** Pregnancy outcomes in patients according to stage of CKD

	Stage 1 (n = 197)	Stage 2 (n = 73)	Stages 3–4 (n = 30)	t^α^	p^α^	t^β^	p^β^	t^γ^	p^γ^
Twin pregnancy [n (%)]^a^	3 (1.5)	1 (1.4)	1 (3.3)	0.23	0.64	0.49	0.61	–	–
Live birth [n (%)]^a^	193 (98)	69 (94.5)	26 (86.7)	1.17	0.28	6.74	< 0.001	0.90	0.34
Live birth infants [n (%)]^b^	196 (98)	70 (94.6)	27 (87.1)	1.17	0.28	6.56	0.01	0.84	0.36
Pregnancy weeks (weeks)^c^	38.0 ± 2.3	38.10 ± 2.3	35.0 ± 2.9	0.47	0.64	3.21	< 0.001	3.31	< 0.001
Mean birth weight (g)^c^	2997 ± 622	3008 ± 555	2303 ± 684	0.15	0.88	6.45	< 0.001	6.86	< 0.001
Delivery by CS (%)^c^	111 (57.5)	44 (63.8)	23 (88.5)	0.82	0.36	9.24	< 0.001	5.54	0.01
HTD in pregnancy [n (%)]^a^	40 (20.3)	16 (21.9)	10 (33.3)	0.08	0.77	2.57	0.11	1.47	0.23
GH [n (%)]^a^	8 (4.1)	2 (2.7)	0	0.02	0.88	–	–	–	–
LPE [n (%)]^a^	4 (2.0)	5 (6.8)	3 (10)	3.13	0.08	6.49	0.01	0.02	0.90
SPE [n (%)]^a^	28 (14.2)	9 (12.3)	7 (23.3)	0.16	0.69	1.03	0.31	1.21	0.27
Fetal and neonatal death [n (%)]^a^	4 (2.0)	4 (5.5)	4 (13.3)	1.17	0.28	6.74	< 0.001	0.90	0.34
Preterm birth [n (%)]^c^	35 (18.1)	9 (13.0)	15 (57.7)	0.94	0.33	20.35	< 0.001	19.9	< 0.001
28–32 weeks [n (%)]	7 (3.6)	2 (2.9)	4 (15.4)	0.01	0.92	4.4	0.04	3.09	0.08
32–34 weeks [n (%)]	8 (4.2)	2 (2.9)	3 (11.5)	0.01	0.92	1.30	0.25	1.36	0.24
LBWI [n (%)]^d^	29 (14.8)	10 (14.3)	12 (44.4)	0.01	0.92	12.0	< 0.001	10.11	< 0.001
VLBWI [n (%)]^d^	7 (3.6)	2 (2.9)	3 (11.1)	0.01	0.92	1.64	0.20	1.29	0.26
SGA [n (%)]^d^	10 (5.1)	4 (5.7)	0	0.01	0.91	–	–	–	–

Values are expressed as mean ± standard deviation or number and percentage

Statistical significance at p = 0.017 after Bonferroni correction for multiple comparisons

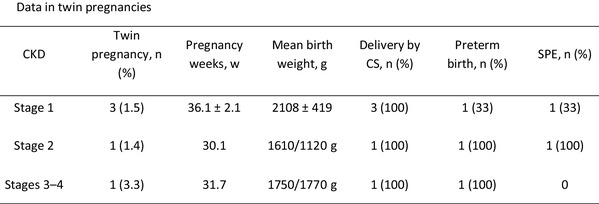

*CKD* chronic kidney disease, *HTD* hypertension disease, *GH* gestational hypertension, *LPE* light preeclampsia, *SPE* severe preeclampsia, *LBWI* low birth weight infants, *VLBWI* very low birth weight infants, *SGA* small for gestational age

^a^Ratio of patients at the corresponding stage

^b^Ratio of fetus at the corresponding stage

^c^Data were available only in live birth patients

^d^Data were available only in live birth infants

^α^CKD stage 2 compared to CKD stage 1

^β^CKD stage 3–4 compared to CKD stage 1

^γ^CKD stage 3–4 compared to CKD stage 2

In patients with stage 1 CKD, there were 3 cases of twin pregnancies. In one pregnancy, complication with severe preeclampsia occurred and the patient delivered at 33^+5^ weeks; the birth weight of the newborns was 1500 and 2200 g, respectively. The other two patients delivered at term. In patients with stage 2 CKD, there was 1 case of a twin pregnancy. Complication with severe preeclampsia occurred and the patient delivered at 30^+1^ weeks; the birth weight of the newborns was 1610 and 1120 g, respectively. In patients with stage 3–4 CKD, there was 1 twin pregnancy. The patient delivered at 31^+5^ weeks due to deterioration of renal function, and the birth weight of the newborns was 1750 and 1770 g, respectively.

### Analysis of risk factors for adverse pregnancy outcomes in pregnant patients with CKD

The incidence of adverse pregnancy outcomes [severe preeclampsia, intrauterine death, neonatal death, early preterm birth (< 34 weeks), very low birth weight infants (< 1500 g)] was 18.3% (36/197) among pregnant patients with stage 1 CKD, 16.4% (12/73) among those with stage 2 CKD and 53.3% (16/30) among those with stage 3–4 CKD. The incidence of adverse pregnancy outcomes was significantly higher among patients with stage 3–4 CKD than among those with stage 1 CKD (p < 0.01). At multivariate logistic regression analysis, proteinuria [odds ratio—OR 1.44 (95% confidence interval 1.17–1.77), p < 0.01], MAP [OR 1.04 (1.01–1.07), p = 0.01] and therapy in early pregnancy (including antihypertensive drugs and immunosuppressive agents) [OR 2.09 (1.30–6.45), p < 0.01] were significant risk factors for adverse pregnancy outcomes (Table 3).

**Table 3:** Univariate and multivariate logistic regression analysis of factors at baseline influencing adverse pregnancy outcomes [severe preeclampsia, intrauterine death, neonatal death, early preterm birth (< 34 weeks), and very low birth weight infants (< 1500 g)]

Baseline characteristics	Univariate			Multivariate		
	OR	95% CI	p	OR	95% CI	p
Age (per year)	0.95	0.88–1.02	0.19			
BMI (per 1 kg/m^2^)	1.04	0.98–1.10	0.17	1.05	0.98–1.11	0.18
CKD						
Stage 1	1	Reference		–		
Stage 2	0.88	0.43–1.80	0.73	0.76	0.35–1.65	0.48
Stages 3–4	5.11	2.29–11.41	< 0.001	1.84	0.68–4.99	0.23
Proteinuria (per 1 g/day)	1.56	1.30–1.87	< 0.001	1.44	1.17–1.77	< 0.001
MAP (per 10 mmHg)	1.05	1.02–1.08	< 0.001	1.04	1.01–1.07	0.01
Therapy in early pregnancy (yes or no)	4.8	2.35–9.79	< 0.001	2.90	1.30–6.45	< 0.001

*OR* odds ratio, *CI* confidence interval, *BMI* body mass index, *CKD* chronic kidney disease, *MAP* mean arterial pressure

### Postpartum renal function of patients with different stages of CKD

The mean postpartum follow-up time of the included patients with different stages of CKD was 49.0 ± 33.1 months. A total of 263 cases were followed up, and the loss to follow-up rate was 12.3%. During the follow-up period, no patients with stage 1 or 2 CKD during pregnancy experienced ESRD or started regular dialysis treatment. Among patients with stage 3 CKD, 2 progressed to ESRD during pregnancy, and 3 progressed to ESRD at 74, 48, and 12 months after delivery. Two of these 5 patients started regular dialysis treatment during the follow-up period. The proteinuria during early pregnancy was higher than 2 g/24 h for all 5 of these patients. Among the patients with stage 4 CKD, 2 terminated the pregnancy in the 2nd trimester due to renal failure; then, regular dialysis was started. Another patient had renal failure 6 months after delivery and started regular dialysis. The follow-up details of the patients with different stages of CKD are reported in Table 4.

**Table 4:** Renal survival in pregnant patients with CKD

Stage	n	Follow-up population [n (%)]^a^	Follow-up time (months)	ESRD [n (%)]^b^	New-onset HT [n (%)]^b^
1	197	167 (84.8)	52.4 ± 33.1	0	8 (4.8)
2	73	70 (95.9)	44.4 ± 28.4	0	5 (7.1)
3	25	21 (84)	42.2 ± 42.2	5 (23.8)	3 (14.3)
4	5	5 (100)	16.4 ± 17.9	3 (60)	0
Total	300	263 (87.7)	49.0 ± 33.1	19 (7.2)	16 (6.1)

*CKD* chronic kidney disease, *ESRD* end stage renal disease, *HT* hypertension

^a^Ratio of patients at the corresponding stage;

^b^Ratio of patients in follow-up population at the corresponding stage

### Effects of pregnancy on renal function in patients with stage 3–4 CKD

**Fig. 1:**
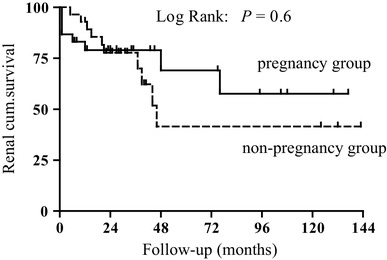
Renal survival in groups of pregnant and non-pregnant women with stages 3–4 CKD

To better evaluate the effects of pregnancy on renal function in patients with stage 3–4 CKD, 28 non-pregnant patients with stage 3–4 CKD were selected as the control group and were matched for age, gender and baseline Scr, 24-h proteinuria, and MAP. In the control group, 9 patients progressed to ESRD during the follow-up period. Multivariate analysis using the Cox regression model showed that the baseline Scr levels and proteinuria were risk factors for renal function deterioration in patients with stage 3–4 CKD, while pregnancy itself did not increase the risk of renal function deterioration. The results of the analyses of risk factors for renal function deterioration in patients with stage 3–4 CKD are presented in Table 5, and the maintenance of renal function based on pregnancy is shown in Fig. 1.

**Table 5:** Risk factors for renal survival in pregnant patients with CKD (stages 3–4)

Risk factors	OR	95% CI	p
Age (per year)	1.006	0.899–1.126	0.915
Serum creatinine (per 20 µmol/l)	1.005	1.002–1.008	0.001
Proteinuria (per 1 g/day)	1.293	1.030–1.623	0.027
MAP (per 10 mmHg)	0.992	0.95–1.036	0.713
Pregnancy	0.815	0.285–2.328	0.703

*CKD* chronic kidney disease, *MAP* mean arterial pressure

## Discussion

With the increasing incidence of CKD in women of childbearing age [3, 4], there is an increasing number of patients with renal impairment who have a strong desire to become pregnant. Among these patients, some have stage 3–4 CKD and have already progressed to moderate-to-severe renal impairment before pregnancy. Therefore, there is important clinical significance in investigating the clinical outcomes of these patients who have been traditionally regarded as having contraindications to pregnancy, in order to help these patients to decide whether to continue the pregnancy or not.

The pregnancy outcomes of patients with stage 1–2 CKD are acceptable; the mean gestational age at birth is 38 weeks. However, the pregnancy outcomes of stage 3–4 CKD patients with moderate-to-severe renal damage were obviously worse, with an overall live birth rate of only 86.7%.

In this study, we investigated whether the prognosis of pregnancy in patients with CKD could be predicted using clinical factors in early pregnancy. Multivariate regression analyses revealed that even for stage 3–4 CKD patients, kidney disease stage was not necessarily the most important influential factor for adverse pregnancy outcomes. Instead, higher proteinuria and MAP in early pregnancy had a greater influence on pregnancy prognosis. One study that enrolled 49 stage 3–4 CKD patients [14] showed that a 24-h proteinuria > 1 g before pregnancy combined with eGFR < 40 ml/min/1.73 m^2^ was a high-risk factor for a small-for-gestational-age fetus and fetal death. The study by Su et al. [15] showed that for patients with CKD before pregnancy, proteinuria during pregnancy negatively correlated with neonatal birth weight. These findings suggest that in patients with moderate-to-severe renal impairment, pregnancy outcome was largely determined by 24-h proteinuria and baseline blood pressure. Better control of baseline urinary protein and blood pressure in these patients will significantly improve pregnancy outcomes; however, this statement is controversial because the use of angiotensin-converting enzyme inhibitors (ACEIs) during pregnancy is limited due to their teratogenic effects. For patients with proteinuria during pregnancy caused by kidney disease rather than preeclampsia, proteinuria can be controlled through low protein diets in order to improve pregnancy outcomes [16–18].

Many previous studies have confirmed that pregnancy does not have an adverse effect on renal function in CKD patients with essentially normal renal function. However, it remains controversial whether pregnancy accelerates the deterioration of renal function in patients with moderate to severe renal impairment before pregnancy. Imbasciati et al. [14] analyzed the effects of pregnancy on the renal function of 49 patients with stage 3–4 CKD and showed that the rate of GFR decline did not obviously change before pregnancy and after childbirth, but for patients with an eGFR < 40 ml/min/1.73 m^2^ and a 24-h proteinuria ˃ 1 g, the rate of GFR decline after childbirth significantly increased. In this study, we followed up patients with different stages of CKD for an average of 49 months, and almost 30% of stage 3–4 CKD patients developed renal failure during this period. Multivariate analysis using the Cox regression model showed that higher Scr levels and higher proteinuria were major risk factors for adverse kidney outcomes, while pregnancy itself did not have a significant negative effect on renal function.

This study has some limitations. First, there were relatively few stage 3–4 CKD patients and, therefore, some risk factors such as the type of CKD could not be analyzed. However, given that patients with severe renal impairment have decreased fertility and that many of those who do become pregnant worry about their kidney disease status and choose artificial abortion in early pregnancy, large-scale, multicenter prospective studies need to be designed to investigate the pregnancy and kidney outcomes of this patient population. Second, most patients in our study had IgA nephropathy, and since the pregnancy outcomes of patients with IgA nephropathy is peculiar [19], this could have been a potential bias of this study. Third, the postpartum follow-up period for patients with CKD was short, and the effects of pregnancy and pregnancy complications such as preeclampsia on the long-term renal function of these patients could not be comprehensively evaluated.

In summary, in this study, we found that the risk of adverse pregnancy outcomes was significantly higher among patients with stage 3–4 CKD than among those with stage 1–2 CKD. However, this risk is mainly associated with proteinuria and hypertension during pregnancy. Pregnancy itself did not seem to accelerate kidney disease progression in patients with stage 3–4 CKD.
